# Multidosing Intramuscular Administration of Methotrexate in Interstitial Pregnancy With Very High Levels of β-hCG: A Case Report and Review of the Literature

**DOI:** 10.3389/fendo.2018.00363

**Published:** 2018-07-10

**Authors:** Valeria Conti, Giovanni Luciano, Giovanni Pecoraro, Roberto Iovieno, Amelia Filippelli, Maurizio Guida

**Affiliations:** ^1^Department of Medicine, Surgery and Dentistry “Scuola Medica Salernitana”, University of Salerno, Fisciano, Italy; ^2^Unit of Gynecology and Obstetrics of “San Giovanni di Dio e Ruggi d'Aragona”, University Hospital, Salerno, Italy

**Keywords:** ectopic pregnancy, b-hCG, interstitial preganancy, treatment scheme, conservative management

## Abstract

Ectopic pregnancy (EP) is the implantation of an embryo outside the endometrial cavity of the uterus. Signs and symptoms of EP may arise between the 6th and the 8th week of gestation and include vaginal bleeding, lower abdominal and pelvic pain. Frequently EPs implant in the fallopian tubes. A rare EP is the interstitial pregnancy, a life-threatening condition being responsible for nearly 20% of all deaths caused by EPs. Because of its unique location, the diagnosis is difficult and based on signs and specific criteria together with measuring of serum β-hCG. Usually, EP is treated by surgical approach, which is associated with increased morbidity, decreased fertility and increased likelihood of hysterectomy and uterine rupture in a subsequent pregnancy. Early diagnosis is crucial to life saving and allowing alternative therapeutic interventions such as pharmacological treatments. Methotrexate (MTX) represents the mainstay therapy. There is no standard care for the interstitial pregnancy for what concerns either surgical or pharmacological approaches. We reported a case of a 36-year-old woman admitted to the Hospital of Salerno-Italy with a value of serum β-hCG of 35,993 IU/L. Transvaginal ultrasonography revealed an empty uterine cavity and a mass of 35.7 mm in diameter characterized by a hypoechoic central area. The patient was in stable haemodynamic condition and no haematologic, renal and hepatic impairments were recorded. Despite the high serum β-hCG levels, a pharmacological approach was preferred to a surgical one. The patient was treated with intramuscular administration of MTX in daily dose of 1 mg/Kg alternated with 0.1 mg/kg folinic acid for 5 days. The patient remained hospitalized for 20 days and no side effects were reported. The decrease of the serum β-hCG was monitored and more than 15% reduction was detected between the 4th and the 7th day after the beginning of the treatment. The serum β-hCG became undetectable 35 days after. A multidosing intramuscular administration of MTX was effective and safe even in the presence of very high serum β-hCG levels. Together with similar cases reported in literature, the present results can contribute to improve the decision making in the treatment of the interstitial pregnancy.

## Introduction

Ectopic pregnancy (EP) is the implantation of a gestational sac outside the endometrial cavity of the uterus that accounts for approximately 2% of all pregnancies (1). Risk factors for EP include a history of previous EP or pelvic inflammatory disease, the use of assisted reproductive technology and tubal surgery (2). Signs and symptoms of EP may arise between the 6th and the 8th week of gestation and include vaginal bleeding, sudden lower abdominal pain, pelvic pain, tender cervix, adnexal mass or adnexal tenderness (1).

More than 95% of EPs implant in the fallopian tube and this localization is followed by those in the fimbria (11%) and the isthmus (12%) (3).

A rare EP is the interstitial pregnancy, which occurs with a low frequency (1–6%) but it is a life-threatening condition being responsible for nearly 20% of all deaths caused by EPs (4). In the interstitial pregnancy the embryo localizes in the interstitium, the most proximal part of the fallopian tubes surrounded by the myometrium (3).

Besides the aforementioned risk factors, the ipsilateral salpingectomy predisposes to the interstitial pregnancy (5).

Because of its unique location, the interstitial pregnancy can be difficult to diagnose. The diagnosis is based on several signs checked on ultrasonography according to specific criteria together with measuring of serum β-human chorionic gonadotropin (β-hCG) (6).

Despite there is no general agreement about the most appropriate surgical approach, EP has been normally treated with hysterectomy or cornual resection by means of laparotomy or laparoscopy (7).

Importantly, surgery is associated with increased morbidity, decreased fertility and increased likelihood of hysterectomy and uterine rupture in a subsequent pregnancy (8). Moreover, surgical approach suffers from risks associated to anesthetic and complications such as severe blood loss, potentially requiring transfusion.

No doubt, however, early diagnosis is crucial to life saving and may decrease the number of surgical procedures allowing alternative therapeutic interventions, such as pharmacological treatments.

Among the authorized drugs, methotrexate (MTX) represents the mainstay drug therapy. MTX is a folic acid antagonist, which inhibits the enzyme dihydrofolate reductase, thereby interfering with DNA synthesis in rapidly dividing the cells forming the trophoblast (9, 10).

It is necessary to consider also the MTX toxicity. However, the life threatening complications following MTX administered for the treatment of EPs are reported rarely (11–14). For instance, Dasari et al. described a case of a 25-year-old woman who was decided to treat by “expectant therapy” but, because of misinterpretation, received medical therapy with multidose MTX (13). The woman developed toxicity with severe bone marrow depression leading to septicemia. Among the factors possibly responsible for such MTX toxicity, the authors suggested the administration of too high dosage with respect to the patient's body weight, MTX hypersensitivity, and genetic polymorphisms such as MTHFR 677TT associated to a decreased MTX clearance. Nonetheless, unexpected toxicity with MTX should be considered during either single or multiple dosing administration.

Here, we describe a case of interstitial pregnancy treated with MTX and we make a narrative review of the available data in this field.

## Case presentation

A 36-year-old Italian woman, gravida 3 para 1, was admitted to the emergency department of the University Hospital “San Giovanni di Dio e Ruggi d'Aragona,” Salerno-Italy with a history of declared 5 weeks amenorrhea and lower abdominal pain.

At the age of 32, she underwent conization for cervical intraepithelial neoplasia, Human Papilloma Virus (HPV) positive. At hysterosalpingography, tubes were not occluded.

**Figure 1:**
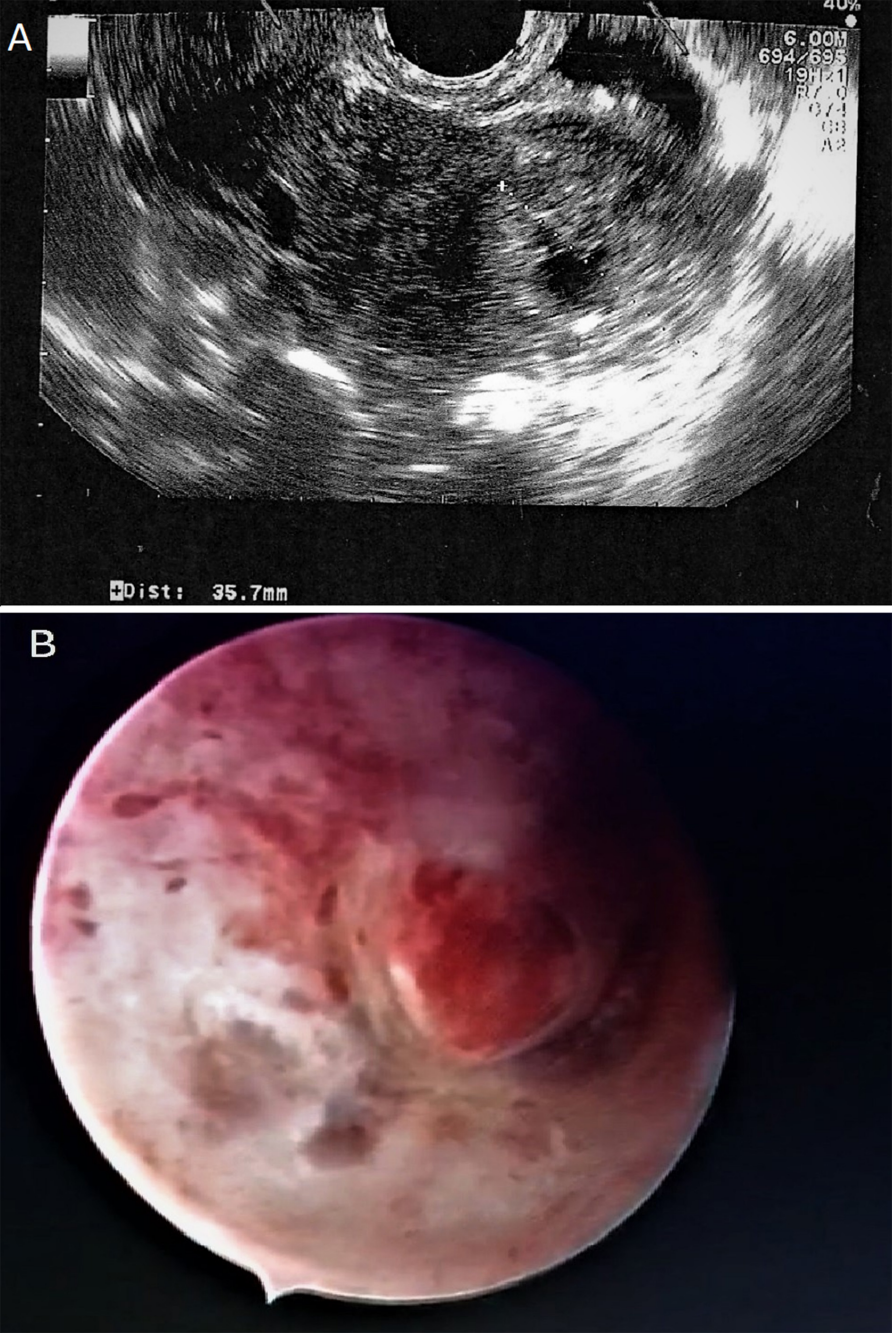
Interstitial pregnancy. **(A)** Coronal view of the uterus on transvaginal ultrasound showing an empty cavity with a mass of 35.7 mm in diameter. **(B)** Hysteroscopy shows the ectopic interstitial pregnancy localized in the left tubaric corner.

At admission, her serum β-hCG was 35,993 IU/L. Transvaginal ultrasonography revealed an empty uterine cavity but a mass of 35.7 mm in diameter characterized by a hypoechoic central area was seen in the interstitium (Figure 1A). Both ovaries appeared normal and there was no free fluid in the Pouch of Douglas. The ectopic interstitial pregnancy localized in the left tubaric corner was confirmed by the hysteroscopy (Figure 1B).

After careful evaluation of the available literature data on the management of EP, a pharmacological approach was preferred to a surgical one. The decision was made taking into account the pros and cons of the surgical approach and in consideration of the ACOG (15) and RCOG (16) guidelines justifying the use of the medical therapy with MTX instead of the surgery.

The administration of MTX was legitimated by the patient's stable haemodynamic condition, and the absence of haematologic, renal and hepatic impairments.

**Figure 2:**
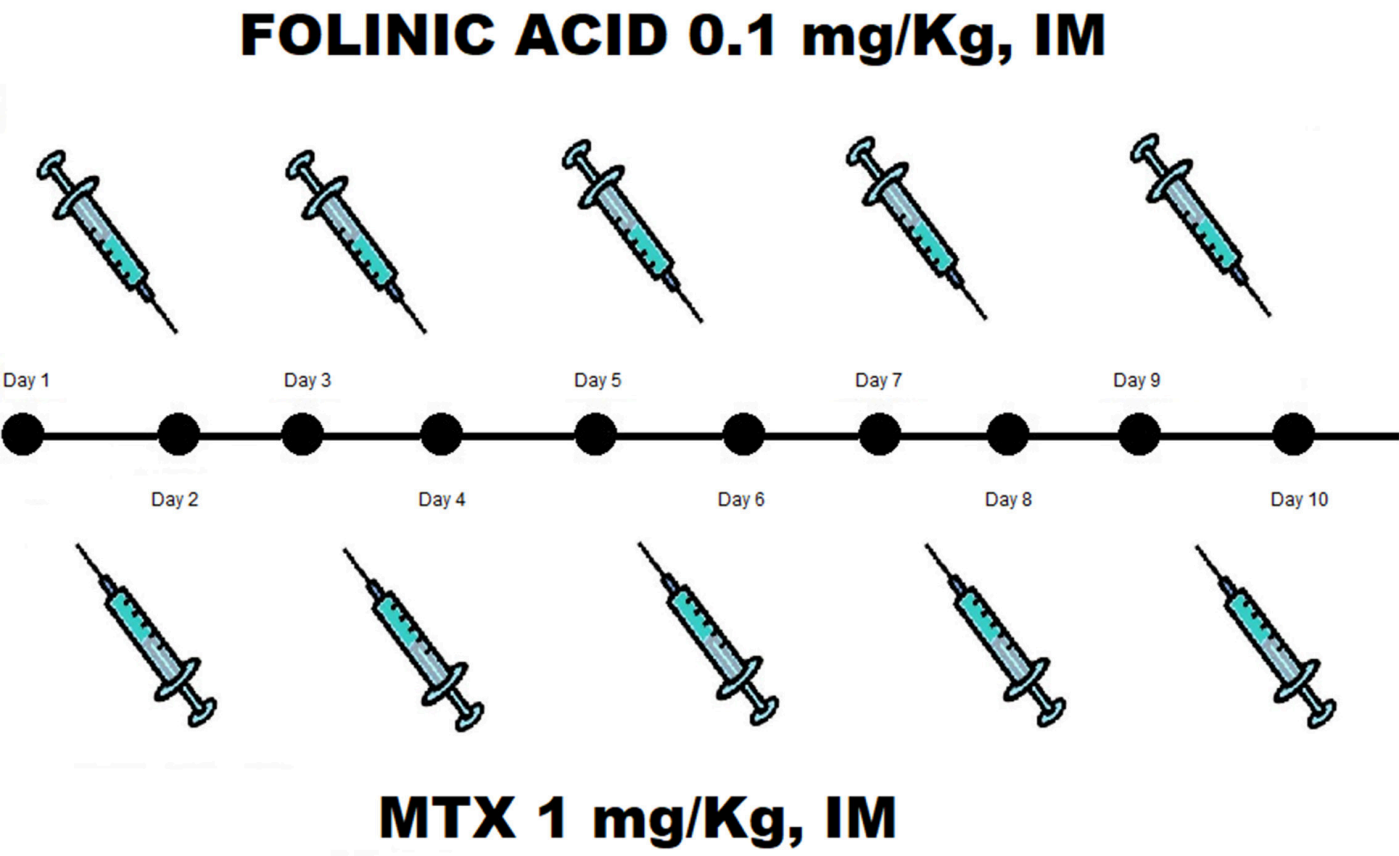
Treatment scheme consists in IM MTX 1 mg/kg alternated with 0.1 mg/kg folinic acid for 5 days.

As the patient showed very high serum β-hCG levels that have been associated to the risk of treatment failure or the need for supplemental MTX dosage, a multiple-dose intramuscular administration of MTX in a daily dose of 1 mg/Kg alternated with 0.1 mg/kg folinic acid for 5 days was preferred to a single dose regimen. The patient provided her written informed consent. The therapeutic scheme was shown in Figure 2.

**Figure 3:**
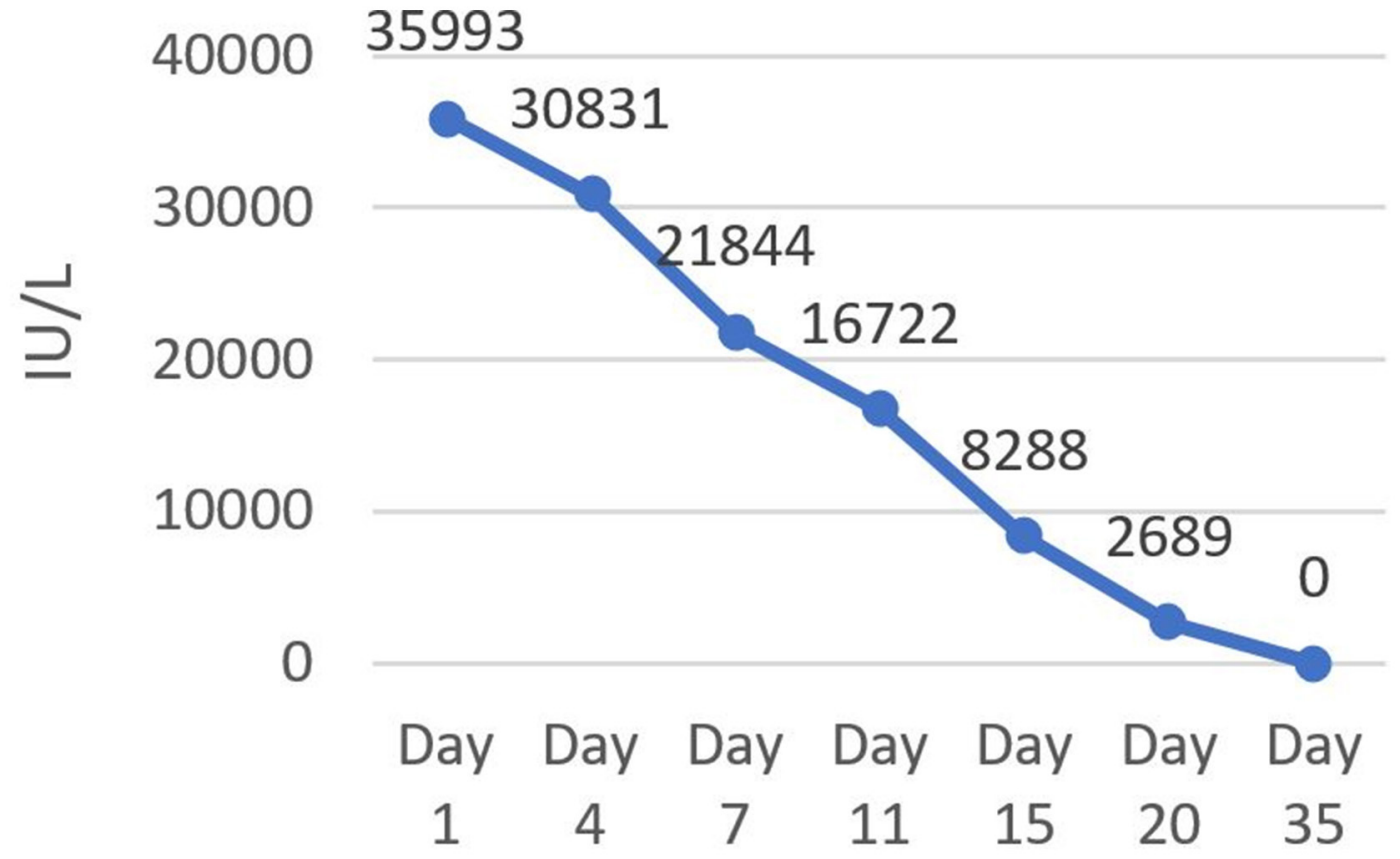
Serum level of β-hCG during hospitalization. A progressive decrease of serum β-hCG was monitored until reduced to zero 35 days after the first MTX injection.

The patient remained hospitalized for 20 days after the first MTX injection. A progressive decrease of the β-hCG serum levels was monitored during hospitalization. Notably, more than β-hCG 15% reduction was detected between the 4th (30,831 IU/L) and the 7th (21,844 IU/L) day after the beginning of the treatment (Figure 3). The serum β-hCG became undetectable 35 days after the first MTX injection.

## Discussion and literature review

Surgery is mandatory for ruptured EPs but in the cases of unruptured EPs, it is possible to opt for a pharmacological approach, especially in the presence of early diagnosis (17).

Pharmacological approach enables to exclude the risks associated to surgery and preserve the woman's fertility (18). Among the licensed drugs, MTX represents the first choice for treatment of all EPs, including interstitial pregnancy.

In the absence of contraindications, the therapy with MTX is authorized in a single intramuscular injection at a dosage of 1 mg/kg or 50 mg/m^2^. Before treatment, laboratory analysis must include the serum β-hCG evaluation, complete blood count, and kidney and liver function tests. The β-hCG evaluation must be repeated on the 4th day. Then, measuring of the serum β-hCG, blood count and kidney and liver functions must be evaluated on the 7th day again. After that, it is necessary to check the serum β-hCG every week until it becomes undetectable. More than 15% decrease between the 4th and the 7th day represents a favorable prognostic marker (19).

A protocol based on a single intramuscular injection of MTX at 50 mg/m^2^ possesses the best risk/benefit ratio (20). However, several reports in literature have described the use of MTX in EPs at different dosages and treatment schemes, differentiated in dependence on time of diagnosis, localization and size of the implanted embryo and presence of signs and symptoms. MTX treatment schemes encompass parenteral injection (either single or multidosing administration) or direct injection into the gestational sac combined with surgery.

In 2016, Marret et al. reviewed a case series representing such variety in the potential uses of MTX into clinical practice for treatment of EPs. These authors reported that the single dose MTX is effective exclusively in tubal pregnancy and the success of the treatment strongly depends on the initial β-hCG level (21, 22).

By comparing surgical treatments to MTX administration, a meta-analysis demonstrated that both single and multiple dosing MTX is less effective than laparoscopic salpingotomy for the treatment of tubal EP. Of note, MTX, either IM single dosage or IM multiple dose regimen used postoperatively, is a good alternative only in selected patients with low serum β-hCG concentrations ranging from < 1,500 IU/L to < 3,000 IU/L. This meta-analysis remarked also that MTX, mainly used in a multidosing regimen, is also less safe than surgery but the effects on the quality of life have not yet been investigated (23).

The use of MTX as an adjuvant treatment alongside a conservative surgery has also been reported with two possible approaches. One of them is the prophylactic use of MTX while the second one consists of IM MTX administered only after salpingotomy failure. In both the approaches MTX seems to reduce the occurrence of secondary tubal rupture and surgical reinterventions. However, no randomized trials have been carried out in this field (24, 25).

Marret et al. brushed up the selection criteria guiding the choice of the most appropriate therapeutic approach in EPs, underlining that it is very important to take into account the symptomatology, the localization and the size of the gestational sac, the patients' history and the β-hCG serum levels both at baseline and during the therapy. The absence of haemodynamic instability and other complications remains the most important selection criterion to guide the choice of treatment (21).

The published data on the management and treatment of interstitial or cervical pregnancy, two EPs mostly associated to morbidity and mortality, indicate MTX as the backbone pharmacological agent used either by IM or by *in situ* injection.

Some authors have reported clinical case of cervical pregnancy treated with local MTX injection under transvaginal ultrasound guidance. A single local MTX injection was found to be effective and safe (26). Notably, Jeng et al. described sixteen cases of EPs in the absence of hemodynamic instability with β-hCG ranging from 2,765 to 18,648 IU/L, managed through transvaginal intra-amniotic and intrachorionic injection of 50 mg MTX under ultrasound guidance (27). The authors concluded that the cervical pregnancy can be successfully managed without surgical intervention by a local injection of MTX, preserving the fertility and favoring the possibility of subsequent uneventful pregnancies (26, 27).

With regard of interstitial pregnancy, Corioni et al. recently reported a case successfully treated with a single-dose of systemic MTX. The case concerned a 36-year-old Chinese woman, who was admitted to the University Hospital in Florence-Italy at nearly 6 weeks of amenorrhea for a suspected EP. The patient did not refer any abdominal-pelvic symptom but a mild vaginal bleeding was noted. At the time of admission, serum level of β-hCG was 8,681 IU/L (28).

In the case that serum β-hCG levels are particularly high, the management of EPs becomes more complicated. In fact, in 1999 Hafner et al. had already described the case of a woman with very high β-hCG value (i.e., 41,150 IU/L) treated by single dose of IM MTX, but in that case the therapy failed and the woman underwent laparotomy and cornual resection (29).

To the best of our knowledge, only one study reported a case of a 29-year-old woman with high levels of β-hCG (i.e., 31,381 IU/L) successfully treated with a single dose IM MTX. However, in this case 94 days were necessary to reduce to zero the β-hCG levels (30), contrary to one of the most important criteria of the conservative treatment for EP that requires the raise of the undetectable β-hCG values in the least time possible (31).

Available literature data describing different managements of EPs are listed in Table 1. Such studies demonstrated that MTX could be effective both in terms of efficacy and safety for a conservative treatment of EPs.

**Table 1:** Case reports on Treatment of Ectopic Pregnancies between 2004 and 2016.

**References**	**n. of cases**	**Type of EPs**	**Risk factors**	**Initial β-hCG (IU/L)**	**Management**	**Time until β-hCG undetectable (days)**	**Outcomes**
30	20	IP	None/tubal disease/PID/ART/IUCD	From 32 to 31,381	IM MTX-1 dose/IM MTX-2 dose/Laparotomy/Expectant	From 15 to 94	Complete resolution without the need of surgical intervention (94%)
32	1	IP	n/a	167,420	100 mg MTX injection ultrasound-guided after a failed response to 3-dose IM 100 mg MTX	49	Complete resolution
33	2	IP	History of salpingectomy History of miscarriage	40,000 and 3,700	Salpingocentesis followed by MTX instillation (50 mg/m^2^) in combination with oral mifepristone (200 mg)	38	Complete resolution
34	8	5 IP 3 caesarean scar	n/a	From 2,458 to 48,550	Combination of IM MTX 1–2 doses (50 mg/m2) with seven once daily doses of oral gefitinib (250 mg)	From 25 to 196	Complete resolution
35	17	IP	Previous EP/ART/PID	15763.8 ± 25147.1	IM MTX 1 mg/kg/day x4 alternating with folinic acid 0,1 mg/kg/or IM MTX 50 mg/m^2^/or uterine artery MTX injection followed by uterine artery embolization	n/a	Complete resolution (70.5%); requirement of a second-line treatment (20.5%)
36	14	IP	Uterine myomas/Pelvic adhesions/high BMI	≤ 5,000	Transabdominal ultrasound-guided injection of MTX (25 mg)	Max 60	Complete resolution
28	1	IP	n/a	8,681	A single-dose of systemic methotrexate (IM MTX 1 mg/kg)	60	Complete resolution
37	33	IP	Previous EP/Saplpngectomy/ART	From 230 to 106,634	A bolus dose of methotrexate 100 mg/200 mg of methotrexate infusion over 12 h	From 19 to 129	Complete resolution (93.9%)
38	394	Tubal	n/a	2,116 ± 3,157 vs. 4,178 ± 3,422	IM MTX-1 dose (50 mg/m^2^)	n/a	Complete resolution (84.6%); requirement of surgery due to treatment failure (15.36%)

*IP, interstitial pregnancy; n/a, not available; PID, pelvic inflammatory disease; ART, assisted reproductive techniques; IUCD, intrauterine contraceptive device*.

Focusing on the interstitial pregnancy, our case report is the first describing the effectiveness of multidosing systemic injections of MTX, achieved quickly in a woman with a very high serum level of β-hCG.

## Conclusion

The administration of methotrexate for treatment of ectopic pregnancy, including the interstitial pregnancy, has been recognized as a feasible therapeutic approach. However, a unanimous consensus has not yet been reached, especially on the drug dosage and treatment schemes that should be used into clinical practice.

Besides the absence of haemodynamic instability, one of the most important criteria is that the β-hCG levels should reduce to zero in the least time possible.

Here, we have shown that a multidosing intramuscular administration of methotrexate was effective and safe, suggesting that such medical approach is a valid alternative to surgery for the treatment of interstitial pregnancy even in the presence of very high serum β-hCG levels.

No doubt, a single case is not sufficient to claim that the medical approach is preferable to the surgical one.

However, together with similar cases already reported in literature, the present results can contribute to improve the decision making in the treatment of the interstitial pregnancy.

## Ethics statement

This is a case report with review of literature for which the patient signed the Informed Consent, providing her approval.

## Author contributions

All coauthors read and met Frontiers in Endocrinology criteria for authorship. VC and GL collect the data and wrote the manuscript, AF and MG directed the work and critically reviewed the manuscript, GP and RI were in charge of the patient and checked the final version of the paper.

### Conflict of interest statement

The authors declare that the research was conducted in the absence of any commercial or financial relationships that could be construed as a potential conflict of interest.
